# A quantitative assessment of natural and anthropogenic effects on the occurrence of high air pollution loading in Dhaka and neighboring cities and health consequences

**DOI:** 10.1007/s10661-023-12046-3

**Published:** 2023-11-22

**Authors:** Riaz Hossain Khan, Zahidul Quayyum, Shahanaj Rahman

**Affiliations:** 1 BRAC James P Grant School of Public Health, BRAC University, Dhaka, 1213 Bangladesh; 2 Department of Environment, Sher-E-Bangla Nagar, Dhaka, 1207 Bangladesh

**Keywords:** Air quality standard, Climate change, Hazard quotient, Hydro-meteorology, Land use

## Abstract

Although existing studies mainly focused on the air quality status in Bangladesh, quantifying the natural and manmade effects, the frequency of high pollution levels, and the associated health risks remained beyond detailed investigation. Air quality and meteorological data from the Department of Environment for 2012–2019 were analyzed, attempting to answer those questions. Cluster analysis of PM_2.5_, PM_10_, and gaseous pollutants implied that Dhaka and neighboring cities, Narayangonj and Gazipur, are from similar sources compared to the other major cities in the country. Apart from the transboundary sources, land use types and climate parameters unevenly affected local pollution loadings across city domains. The particulate concentrations persistently remained above the national standard for almost half the year, with the peaks during the dry months. Even though nitrogen oxides remained high in all three cities, other gaseous pollutants, such as CO and O_3_, except SO_2_, showed elevated concentrations solely in Dhaka city. Concentrations of gaseous pollutants in Dhaka vary spatially, but no statistical differences could be discerned between the working days and holidays. Frequency analysis results and hazard quotients revealed the likelihood of adverse health outcomes in Narayangonj ensuing from particulate exposures surpasses the other cities for different age, gender, and occupation groups. Nonetheless, school-aged children and construction workers were most at risk from chronic exposure to gaseous pollutants mostly in Dhaka. One limitation of this study was that the routine air quality monitoring happens just from five sites, making the evidence-based study concerning health outcomes quite challenging.

## Introduction

Air pollution is a major concern aggravating the public health burden in many large cities globally. The risks of the criteria air pollutants for respiratory, cardio-, and cerebrovascular diseases were extensively studied in past literature (Zhong et al., 2019). For instance, significant positive correlations between short-term daily and seasonal air pollution from particulate matter (PM_2·5_, PM_10_), sulfur dioxide (SO_2_), and ozone (O_3_) concentrations and hospitalizations with cardiovascular and respiratory illness were reported in China, Iran, Taiwan, Pakistan, Nepal, Myanmar, Turkey, several European cities, and part of the USA (An et al., 2021; Çapraz & Deniz, 2021; Chen et al., 2020; Dehghan et al., 2020, Du et al., 2019; Ikram et al., 2015; Jiang et al., 2020; Liang et al., 2019; Maharjan et al., 2022; Schwartz et al., 2002; Wu et al., 2022a, b; Yang et al., 2021; Zeng et al., 2020). Results showed that females and elder groups (65 years or older) are highly vulnerable to hospitalization risks (Begum & Hopke, 2019). On the other hand, Cesari et al. (2016) reported a significant positive association between exposure to high PM_2.5_ and hospitalizations for pneumonia and asthma in children (below 10 years) living in Taubate. A study in three major cities in Pakistan, Nepal, and Myanmar reported that street workers and construction laborers are highly vulnerable to particulate matter and vehicle exhaust emissions (Maharjan et al., 2022). Although the global burden of chronic obstructive pulmonary disease (COPD) deaths from household air pollution declined in general, an inverse relation was noticed in terms of ambient particulate matter (APM) contributions at low socio-demographic index (SDI) regions where the household air pollution (HAP) is still above standard (Wu et al., 2022a, b). According to Zhong et al. (2019), high O_3_ and nitrogen dioxides (NO_2_) might have more pronounced health impacts than particulate matter. O_3_ generally shows diurnal variations. Han et al. (2016) examined and reported that the association is noticeable when the O_3_ level remains at maximum concentration level and the diurnal variability is significant.

Like many densely populated cities in low- and middle-income countries, ambient concentrations of particulate matter and nitrogen oxides often exceed the national ambient air quality standard (NAAQS) in several rapidly developed and highly urbanized cities in Bangladesh (Begum et al., 2013). Among those, the capital city Dhaka and two adjoining cities called Narayangonj and Gazipur show significant outdoor ambient air pollution levels resulting from local sources such as high construction activities, fossil fuel burning, biomass burning, and brick Kiln operations, as well as transboundary sources, during the dry months (Begum et al., 2011a; Begum et al., 2013; Begum et al., 2016; Rahman et al., 2019; Rana & Khan, 2020). Rapid changes in land use characteristics resulting from high population expansion altered air quality status within a decadal-scale timeframe. Considering all the above, Dhaka, Narayangonj, and Gazipur were considered ideal candidate sites for this study.

In Dhaka, black carbon accounts for about 50% of the total fine PM mass. About 90% of PM_2.5_ mass comes from anthropogenic sources such as soil dust, motor vehicle exhaust, and industrial emission including brick kiln operation, diesel-powered generators, and biomass burning sources (Begum et al., 2011b, c; Begum et al., 2014; Begum & Hopke, 2019; Rahman et al., 2020; Rahman et al., 2021). A large number of existing motor vehicles on the roads and a sharp increase in motorbikes result in an uncontrolled emission of carbon monoxide (CO), nitrogen oxides, and SO_2_ from using inferior-quality fuels, unfit vehicles, and mud and dust from wheels. A large number of small to mega construction activities such as road and building that comprise road cutting, transportation of construction materials, and burning of pitches mainly release PM_10_, polycyclic aromatic hydrocarbons (PAH_s_), volatile organic carbons (VOCs), asbestos, and gases such as CO, CO_2_, and nitrogen oxides throughout the year. In general, the majority of the construction takes place during the dry months. Other significant air pollution sources are the “brick kilns,” primarily in Narayangonj, Gazipur, and Savar (Begum et al., 2014). Based on the available data from the Department of Environment, as of 2022, 284 out of 284, 282 out of 294, and 148 out of 148 brick kilns in Dhaka, Narayangonj, and Gazipur districts have adopted improved technologies for brick manufacturing. However, most of them have included zigzag/improved zigzag with a small percentage (10%, 2%, and 8% in Dhaka, Narayangonj, and Gazipur districts, respectively) of hybrid Hoffman, automatic/tunnel kiln, and other improved environmentally friendly technologies. On the other hand, only 59% of brick kilns in Dhaka, 71% in Narayangonj, and 58% in Gazipur districts have environmental clearance from the Department of Environment. Most of those brick kilns are operational during the winter (October to March), emitting a large amount of SO_2_, CO, black carbon, and CO_2_, bringing the pollutants to Dhaka city through wind transport processes. The concentration level of the different criteria pollutants significantly varies depending on land use activities ranging from daily, seasonal, to decadal-scale time frames.

A study in Tehran found that temperature had a significant role in the daily and seasonal variability of particulate concentrations. On the other hand, solar radiation had important contributions to the SO_2_ and O_3_ concentration levels (Yousefan et al., 2020). In Bangladesh, the effects of climate change are evidenced mainly by an increase of mean minimum temperature to the extent of about 0.5°C in decadal-scale time and a shift of rainfall peak, including more intense annual cumulative rainfall in recent years. Therefore, along with local anthropogenic contributions, the roles of the meteorological parameters under the changing climate for the variability of the pollution level need a detailed investigation. In the study area, the absence of rainfall, relatively more construction activities, brick kiln operations, and other causal factors result in high pollution levels affecting vulnerable population groups during the dry months (Rahman et al., 2019). Sushan (2021) reported that existing air pollution levels might aggravate incidences and severity of the top ten diseases, including cardiovascular and respiratory illnesses, which generally cause high annual deaths in Bangladesh. Therefore, it is of utmost importance to conduct a comprehensive assessment regarding public health consequences for different ages, genders, and occupation groups to examine the association with the current pollution level in the study areas.

Despite significant advancements in the studies of the air quality status in the study areas, the long-term diurnal variability, quantification of the contributing factors to the variable air pollution levels, and associated human health hazards remained beyond investigation. Therefore, this research sought to answer the following:

- How do the concentrations of the criteria air quality parameters vary in multiple temporal scales ranging from daily to decadal-scale time frames spatially, including the roles of hydrometeorological variables and human-induced activities on the concentration levels of the pollutants?
- What is the probability (frequency) of occurrence of significant high pollution loads in the scale of years?
- Which age, gender, and occupation groups are at higher chronic health risk?

Finally, this study provided insights regarding existing policy and implementation gaps and recommendations for potential solutions to reduce the emissions from anthropogenic sources of pollution.

## Materials and methods

The Department of Environment (DoE), a government organization, operates eleven continuous air monitoring stations (CAMS) in eight different cities in Bangladesh. PM_2.5_, PM_10_, CO, O_3_, NO_2_, and SO_2_ have been identified as criteria air pollutants by the World Health Organization (WHO) (2006) and the US Environmental Protection Agency (USEPA) (2016). Data of those criteria pollutants, including several meteorological parameters (rainfall, wind speed, wind direction, solar radiation, air temperature, barometric pressure) in three stations at Dhaka, one in Narayangonj, and one in Gazipur since 2012, was considered for this study (Department of Environment, 2019). In Dhaka, three CAMS are located in Sher-e-BanglaNagar (CAMS 1) at 23.76 N, 90.39 E, Firmgate (CAMS 2) at 23.76 N, 90.39 E, and Darus-Salam (CAMS 3) at 23.78 N, 90.36 E. The stations at Gazipur (CAMS 4) and Narayangonj (CAMS 5) are at 23.99 N, 90.42 E, and 23.63 N, 90.51 E, respectively. The locations of the continuous air monitoring stations are shown in Fig. 7.

**Fig. 1:**
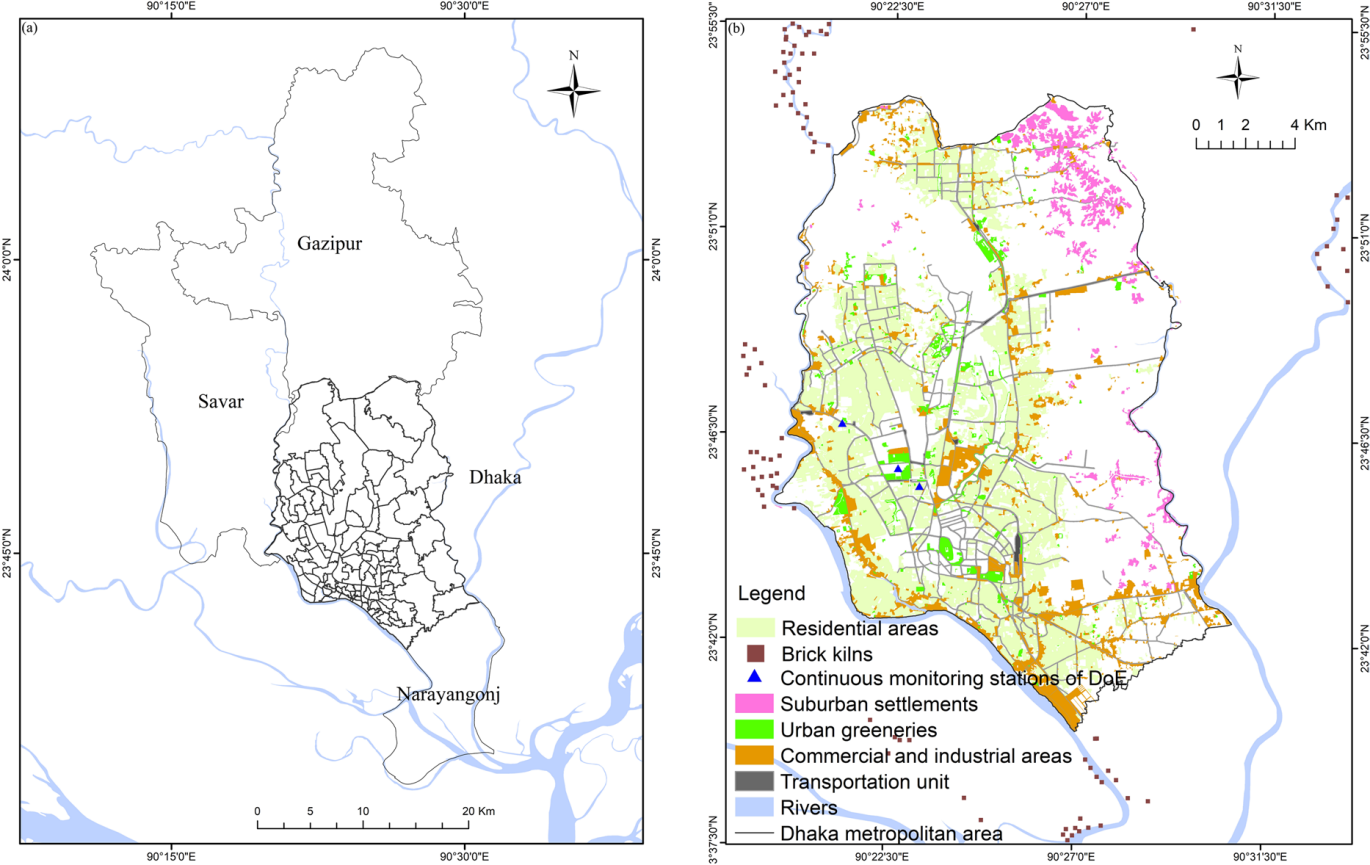
**a** Location map of the study areas and **b** current land use map of Dhaka city

The location map of the study area is shown in Fig. 1a. Dhaka city is mainly comprised of mixed land use types, including the majority of the residential areas towards the western half of the city, the suburban areas located towards the northeastern part, dispersedly located roads, green spaces, industrial and commercial areas, biomass burning, and also a large number of brick kilns at the outer boundaries of Dhaka north and south city corporations (Fig. 1b).

At first, cluster analyses were conducted to assess any similarity in characteristics for the available air quality parameters from the eleven DOE CAMS stations from eight different cities in Bangladesh. Due to high population density and rapid population growth, Dhaka is expanding laterally and partially merged with the neighboring towns. Therefore, data indicates similarities in characteristics among the cities of Dhaka, Narayangonj, and Gazipur. Considering this, the rest of the analyses concentrated mainly on five CAMS at Dhaka, Narayangonj, and Gazipur.

Time series analyses were conducted to assess temporal changes in the daily exceedance rate in seasonal and decadal-scale periods at DOE stations in Dhaka, Narayangonj, and Gazipur. Statistical hypothesis tests were conducted to see differences in concentration levels among those CAMS locations and variations of concentration due to high outdoor activities during working days compared to holidays. At first, quantile–quantile (*Q*–*Q*) normality plots and Shapiro–Wilk test were conducted to check the normality of the data distributions. If the dataset showed normal distribution, a *t*-test was conducted to assess the statistical differences in the mean values between the corresponding groups. On the other hand, for non-normal data, the hypothesis tests were conducted using a permutation or rank-based test depending on the differences in sample medians and any significant outliers that may affect the means. For the permutation test, the data were resampled 1000 times for each group separately and then subtracted the resample means. The mean differences were examined using density plot distributions and normality plots. If a significant difference in the medians from the boxplots and outliers was found to significantly distort the means for both series, a rank-based (Kruskal–Wallis) method was applied to see statistical differences. The maximum diurnal fluctuations were calculated for all criteria pollutants in 2013, 2015, and 2018. Then, polynomial curve fitting was applied for the dataset for given years, and the polynomial order was chosen given that the model provided the highest explanatory power (*R*^2^ values). Finally, the patterns of the curve and the minima and maxima were compared side by side to assess any trends in the magnitude of the daily fluctuations over the years. Then, analysis of variance (ANOVA) was conducted at a 95% confidence interval to estimate the contributions of the hydrometeorological and manmade contributions to the variations of pollution loading for different air quality parameters. The frequency of high pollution levels of air pollutants at different monitoring stations was estimated using Gumbel’s probability estimation method (Mahdi & Cenac, 2004). For the frequency analysis, extreme value effects were carefully removed from the datasets.

The spatial distribution of the non-carcinogenic human health risk factor was mapped based on the estimated hazard quotient (HQ) for various age, gender, and occupation groups. As part of the risk characterization process, the chronic exposure for the criteria pollutants was first assessed using the following rate equation.

Average daily dosage for inhalation of individual pollutants:

1 $$ADD\ \left( in\ \mu g/ kg/ day\right)=\frac{C\times IR\times ED}{BW\times AT}$$

where *C* is the volume of the chemical in ambient air (μg/m^3^), *IR* is the inhalation rate (m^3^/day), *ED* is the period (days) of exposure, *BW* i the body weight (kg) of exposed group, and *AT* is the averaging time (days) (WHO 1999).

The period (days) of exposure (ED) is assessed based on Eq. 2 as follows:

2 $$ED= ET\times EF\times DE$$

where *ET* is the exposure time (h/day), *EF* i the frequency of exposure to the pollutant of interest (days/year), and *DE* is the period (year) of exposure (Embiale et al., 2020; Morakinyo et al., 2017; Morakinyo et al., 2021).

Then, chronic non-carcinogenic health risks were estimated by solving the following equation.

For chronic exposure:

3 $$hazard\ quotient\ (HQ)=\frac{ADD}{RfC}\ \Big)$$

R_f_C approximates the reference concentration level above which continuous inhalation exposure to the human population may cause considerable adverse health outcomes during a lifetime.

According to the USEPA (1989):

- HQ < 1 indicates no risk to human health.
- HQ = 1 indicates marginal risk, indicating that the contaminant is not a potential health risk to a sensitive person.
- HQ > 1 indicates risks to some extent upon exposure to different individuals, adults, and children.

Geospatial maps of the total hazard quotients for significant air pollution parameters were prepared using spatial interpolation for the identified vulnerable population groups.

Finally, existing policies, policy, and implementation gaps and possible solutions were provided for the major air pollution sources across the city.

## Results and discussion

### Clustering based on pollution levels in available DOE CAMS sites at eight different cities in Bangladesh

**Fig. 2:**
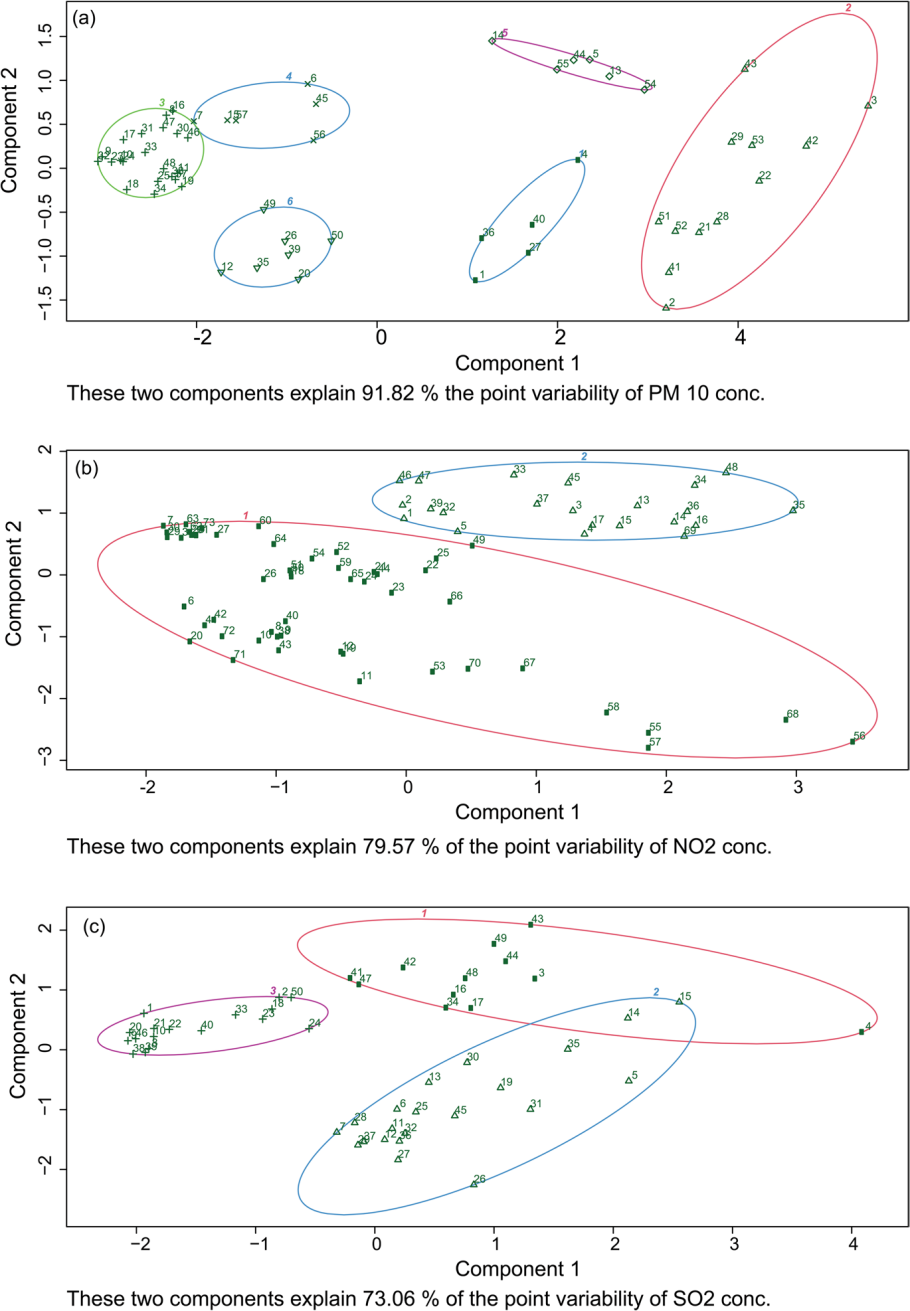
Clustering based on **a** PM_10_, **b** NO_2_, and **c** SO_2_ concentration in different cities in Bangladesh

The pollution level for criteria pollutants showed noticeable spatial differences in eight major cities in Bangladesh. For instance, the data were grouped into six major cluster groups based on particulate concentrations where Dhaka, Narayangonj, and Gazipur were clustered together, indicating similar sources of pollution (Fig. 2a). On the other hand, the gaseous pollutants such as NO, NO_2_, and SO_2_ showed some similarity in different cities (Fig. 2b–c). They were grouped up to a maximum of four cluster groups where the three monitoring stations at Dhaka showed similar characteristics compared to the other CAMS locations.

### Long-term variations in the daily exceedance rate indicate the persistence of high concentration levels of the criteria pollutants in the study areas

**Fig. 3:**
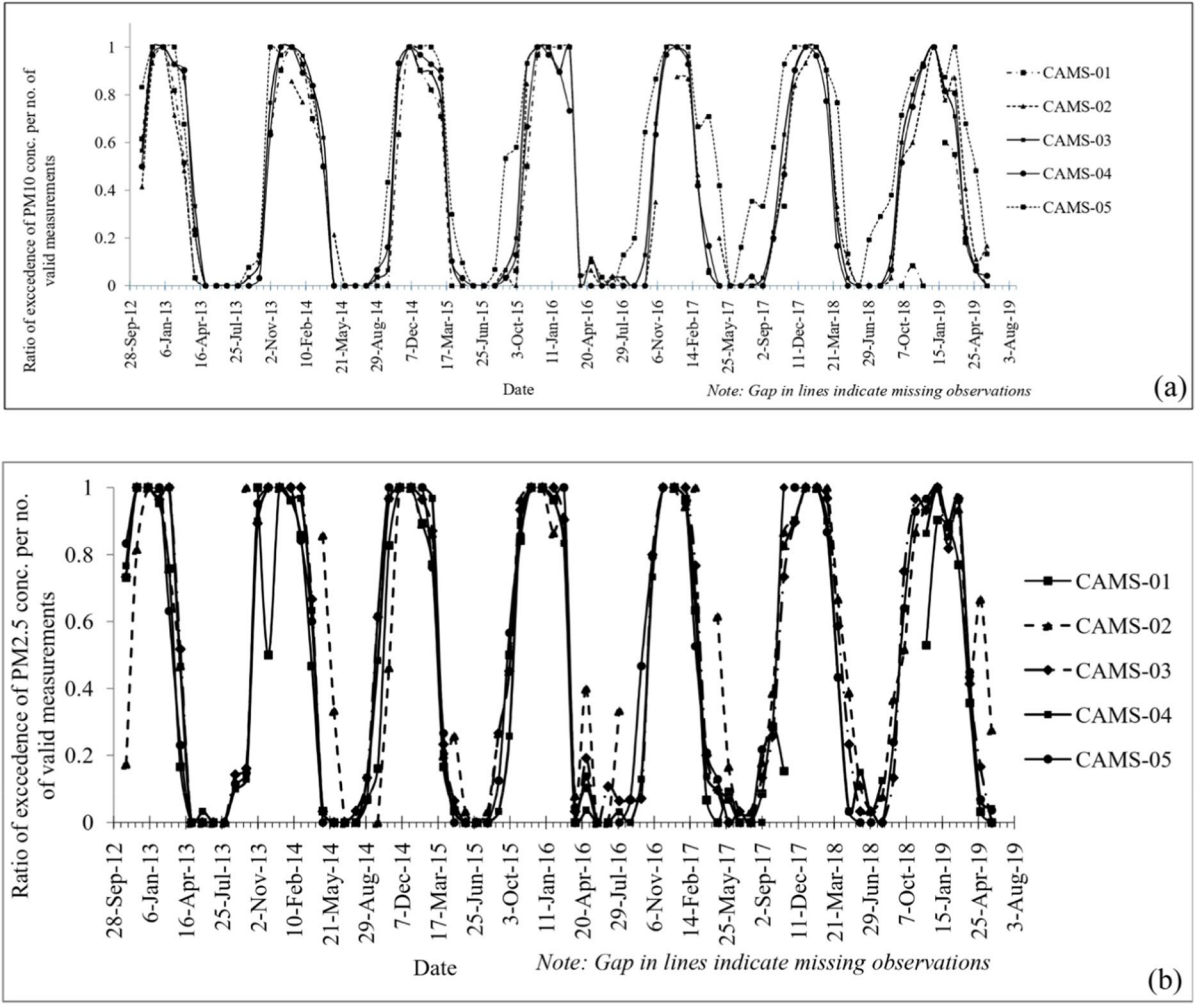
Proportion of the daily exceedance of **a** PM_10_ and **b** PM_2.5_ concentration relative to NAAQS (data from April 2012 to June 2019)

Comparing the ratio of exceedance to the number of valid measurements for the stations at Dhaka, Narayangonj, and Gazipur showed that PM_2.5_ and PM_10_ concentrations remain above the NAAQS for a significant portion (about 6 months) of the year (Fig. 3). In recent years, the annual duration of high pollution levels has become higher for the station at Narayangonj CAMS, which might result from an increase in motor vehicles, construction, and various industrial activities. During the dry season, especially in November through February, the primary wind vector is from west to central to east and south. Therefore, the air quality at those stations, especially in Dhaka and Narayangonj, is also perturbed by many brick kiln operations at the western periphery of the city boundary during the brick manufacture season (mainly in winter) along with the aforementioned local sources.

The main local source of NO_2_ in Dhaka, Narayangonj, and Gazipur is the combustion of fossil fuel, which might be related to the high traffic jams, a significant increase in the number of motorbikes, using inferior-quality fuel, and running unfit vehicles on the roads along with other anthropogenic sources. Although the concentration of NO_2_ often exceeds the NAAQS at all monitoring stations, a noticeable high CO and O_3_ were noticed in Dhaka city only.

Generally, ground-level ozone is created through the interactions of the emitted volatile organic compounds and nitrogen oxides in the presence of heat and sunlight. The major sources of the VOCs and nitrogen oxides in the study areas are the motor vehicles in all cities and partially emitted from the industrial and chemical manufacturing sources. In Dhaka city, the major sources of nitrogen oxides and VOCs are from a large number of vehicles that ultimately significantly contribute to the rise of the O_3_ level. Therefore, the high NO_2_ concentrations may increase public health risks in all cities, for example, an increase in pediatric asthma incidence in those urban areas (Anenberg et al., 2022). However, the other gaseous pollutants except SO_2_ are a potential concern for Dhaka city only.

### Spatial differences in the concentrations of the criteria pollutants among three CAMS stations in Dhaka

Dhaka Sher-e-Bangla Nagar CAMS (CAMS 1) showed significant differences in particulate matter concentration compared to Dhaka Firmgate CAMS (CAMS 2) and Dhaka Darus-Salam CAMS (CAMS 3) (Table 1). However, CAMS 2 and CAMS 3 did not show any noticeable differences. The annual variability of the average PM concentrations is the highest in CAMS 3, and CAMS 1 accounts for the least. On the other hand, for the concentrations of gaseous pollutants, NOx, CO, and O_3_ and all stations significantly differ from one to another in Dhaka (Table 1). Similar to PMs, the concentration of CO is highest in CAMS 3 (Table 1). On the other hand, the maximum concentration of NO was noticed in CAMS 1 compared to the other two stations. The annual variability of O_3_ is significantly higher in CAMS 2 and 3 compared to CAMS1.

**Table 1:** Statistical differences in the concentration of the air quality parameters among the air monitoring stations in Dhaka

Stations	Types of hypothesis test	*P* value of significance	Confidence interval (at 95%)	Significant difference or not
*Statistical differences in PM*_*10*_ *concentrations*				
CAMS 01 vs. CAMS 02	*t*-test	0.0001567 < 0.05	(−54.29727, −17.43616)	Yes
CAMS 02 vs. CAMS 03	Permutation	0.05261 > 0.05	(−14.24507, 14.47179)	No
CAMS 03 vs. CAMS 01	*t*-test	4.323e^−07^ < 0.05	(−65.51957, −29.41983)	Yes
*Statistical differences in PM*_*2.5*_ *concentrations*				
CAMS 01 vs. CAMS 02	*t*-test	0.0007809 < 0.05	(12.54187, 45.79706)	Yes
CAMS 02 vs. CAMS 03	Permutation	0.2401 > 0.05	(−8.837444, 8.573176)	No
CAMS 03 vs. CAMS 01	*t*-test	0.01025 < 0.02	(5.321804, 38.459637)	Yes
*Statistical differences in O*_*3*_*-8-h concentrations*				
CAMS 01 vs. CAMS 02	Kruskal–Wallis	5.908e^−09^ < 0.05		Yes
CAMS 02 vs. CAMS 03	Kruskal–Wallis	2.2e^−16^ < 0.05		Yes
CAMS 03 vs. CAMS 01	Kruskal–Wallis	0.001019 < 0.05		Yes
*Statistical differences in NO*_*x*_ *concentrations*				
CAMS 01 vs. CAMS 02	Kruskal–Wallis	5.908e^−09^ < 0.05		Yes
CAMS 02 vs. CAMS 03	Kruskal–Wallis	2.2e^−16^ < 0.05		Yes
CAMS 03 vs. CAMS 01	Kruskal–Wallis	0.001019 < 0.05		Yes
*Statistical differences in CO-8-h concentrations*				
CAMS 01 vs. CAMS 02	*t*-test	0.2543 7 > 0.02	(−0.1352513, 0.5050405)	No
CAMS 02 vs. CAMS 03	Kruskal–Wallis	2.2e^−16^ < 0.05		Yes
CAMS 03 vs. CAMS 01	Kruskal–Wallis	0.0003412 < 0.05		Yes

### Effect of outdoor activities on the ambient pollution levels during working days and holidays

**Fig. 4:**
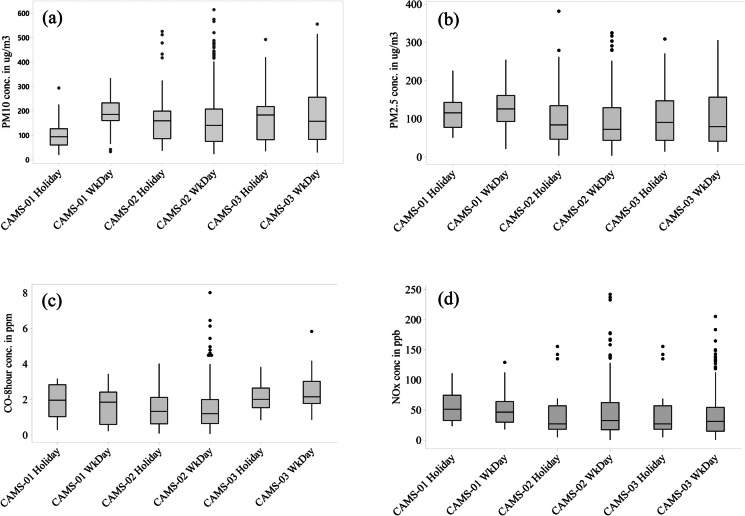
Boxplots show a comparison of the concentration level of different criteria pollutants **a** PM_10_, **b** PM_2.5_, **c** CO, and **d** NO_x_ during weekdays and holidays in Dhaka city

A comparison of the particulates and gaseous concentrations did not show any significant statistical differences during the holidays compared to the working days at most air monitoring locations in Dhaka (Table 2). Only the CAMS station at Sher-e-Bangla Nagar CAMS showed differences in PM_10_ concentration during the weekdays compared to the holidays. Overall, the results indicate that the land use activities contributing to air pollution do not noticeably vary during the holidays. However, the fluctuations of particulate concentrations in the Darus-Salam (CAMS 3) are relatively more significant on weekdays (Fig. 4). Another feature of the Firmgate CAMS (CAMS 2) location is that it showed frequent extreme concentration levels for most of the criteria pollutants during the weekdays (Fig. 4).

**Table 2:** Statistical differences in the concentration of the air quality parameters within the continuous air monitoring stations (weekdays versus holidays) in Dhaka

Stations	Types of hypothesis test	*P* value of significance	Confidence interval (at 95%)	Significant difference or not
*Statistical differences in PM*_*10*_ *concentrations*				
CAMS 01	*t*-test	2.994e^−10^ < 0.05	(−113.03445, −64.57066)	Yes
CAMS 02	Permutation/Kruskal–Wallis	0.624 > 0.05	(−25.80268, 28.37145)	No
CAMS 03	Permutation/Kruskal–Wallis	0.6701 > 0.05	(−26.81381/25.51888)	No
*Statistical differences in PM*_*2.5*_ *concentrations*				
CAMS 01	*t*-test	0.7366 > 0.05	(−41.30002, 29.75567)	No
CAMS 02	Permutation/Kruskal–Wallis	0.624 > 0.05	(−29.62018, 28.79538)	No
CAMS 03	Permutation/Kruskal–Wallis	0.6701 > 0.05	(−25.51069, 27.03191)	No
*Statistical differences in O*_*3*_*-8-h concentrations*				
CAMS 01	Permutation/Kruskal–Wallis	0.147 > 0.05	(−0.6833824, 0.7324258)	No
CAMS 02	Permutation/Kruskal–Wallis	0.8892 > 0.05	(−1.358979, 1.459876)	No
CAMS 03	Permutation/Kruskal–Wallis	0.8664 > 0.05	(−2.430919, 3.236811)	No
*Statistical differences in CO-8-h concentrations*				
CAMS 01	*t*-test	0.3533 > 0.05	(−0.3749642, 0.9936682)	No
CAMS 02	*t*-test	0.5136 > 0.05	(−0.4793874, 0.2418665)	No
CAMS 03	*t*-test	0.09568 > 0.05	(−0.5725649, 0.0481233)	No
*Statistical differences in NO*_*x*_ *concentrations*				
CAMS 01	*t*-test	0.3355 > 0.05	(−8.124937, 22.721350)	No
CAMS 02	Permutation/Kruskal–Wallis	0.8892 > 0.05	(−1.401254/1.452761)	No
CAMS 03	Permutation/Kruskal–Wallis	0.8664 > 0.05	(−2.372265/2.988573)	No

### Year-to-year variations of the daily fluctuations of the concentrations of major air pollutants

**Fig. 5:**
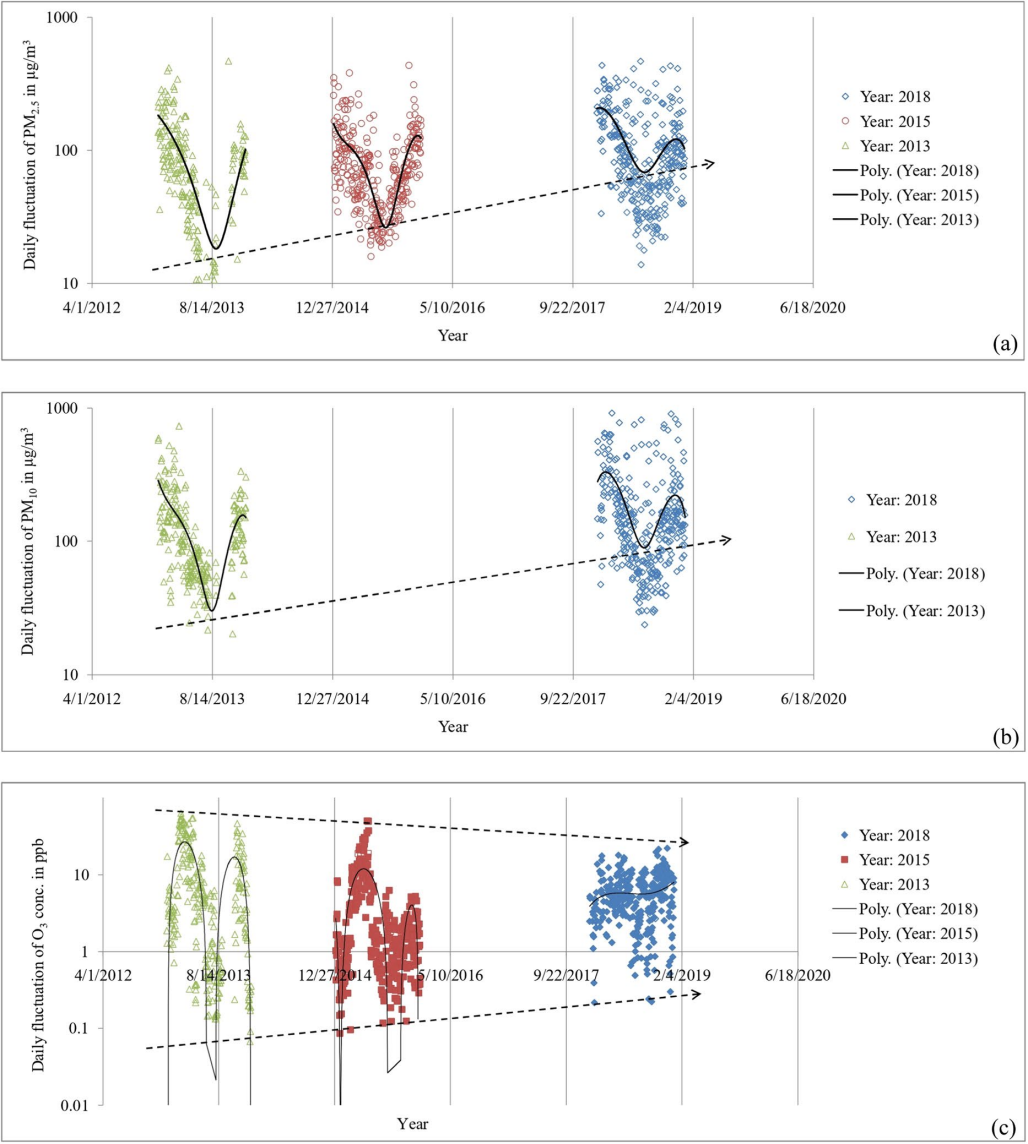
Diurnal variability of **a** PM_2.5_ and **b** PM_10_; **c** 8-h O_3_ concentrations in Dhaka city

We examined maximum daily fluctuations for the criteria pollutants for 2013, 2015, and 2018. The datasets showed increased diurnal variability for PM_2.5_ and PM_10_ in Dhaka (Fig. 5a–b).

The gaseous pollutant, ground-level O_3_, showed a distinctive bimodal peak of the maximum variability once in pre-monsoon (April to May) and the second in the winter season in 2013 (Fig. 5c). Although the dataset in 2015 showed the pre-monsoon peak became predominant, the bimodality was noticeably reduced in 2018. Overall, the annual variability showed gradual decreasing trends over the period. Like the PMs, fluctuations of NO showed a seasonal pattern; overall, the diurnal variability is smaller during monsoon. On the other hand, NO_2_-24h and CO-8h do not show any seasonal patterns of the diurnal variability of the concentrations. Also, no noticeable differences were observed in the diurnal variability of NO, NO_2_, and CO over the years.

### Natural and manmade contributions to the spatial variability of pollutant concentrations

In general, the monsoon in Bangladesh (May to October) is characterized by a relatively higher temperature, high relative humidity, and heavy rainfall, which is quite the opposite during the winter months (December to January). During the monsoon, rainwater washes out, significantly reducing air pollution. Higher concentration of pollutants is observed in the dry winter months, which are related to less rainfall, transportation from transboundary pollution sources, and relatively less dilution of emissions due to the lower wind speed (Begum et al., 2011c). One crucial source of higher gaseous pollutants in Dhaka is partially related to the emission sources from two major industrial and brick kiln areas, i.e., Narayangonj to the south and Gazipur to the north of Dhaka city. During the wet season, the major vector of wind direction is from the south and southeast directions, whereas in the dry season, the predominant wind flow is from the northwest direction. The wind speed is relatively greater during the wet season compared to the dry period.

Overall, the concentration of particulates is negatively related to air temperature (−0.84) and positively related to barometric pressure (+0.8). A strong correlation (+0.85) between PM_2.5_ and PM_10_ suggests a similar source of pollution that includes release from various anthropogenic activities such as vehicle emissions, road dust, construction activities, and brick kiln manufacturing. Although the mean PM_2.5_ and PM_10_ ratio varies significantly (0.05 to 0.85) at different monitoring stations in different seasons in Dhaka, no specific seasonal trends were observed. PM_2.5_ exhibits a positive correlation with NO_x_ that indicates similar pollution sources (e.g., road transportation, including emissions of incomplete combustion of fossil fuels), consistent with Islam et al. (2015) results. Nitrogen oxides negatively correlated with air temperature (−0.43), duration and magnitude of rainfall, and wind speed. As a result, slightly higher concentrations of NO_x_ and PM_2.5_ were noticed during nighttime at most of the monitoring stations during the dry months due to low wind speed and a slight reduction in ambient temperature. Besides, the concentration of ground-level O_3_ is positively related to the concentration of nitrogen oxides (+0.33). Similar to NO_x_, the concentration of SO_2_ is negatively related to air temperature (−0.45) and barometric pressure (−0.68).

ANOVA test results showed that meteorological factors considerably influence the variations in the concentration of the criteria pollutants. Along with rainfall effects, wind speed (6 to 54%) and solar radiation (1 to 7%) played significant roles in the variations of PM levels. Besides, the gaseous pollutants noticeably contribute to the formation of fine particulates. On the other hand, wind speed (4.5 to 13%), air temperature (7 to 21%), relative humidity (about 9%), and barometric pressure (6 to 8%) might have some noticeable control on the variations of ground-level O_3_ concentrations which is consistent with the results from Vasiliauskienė et al. (2021). Among the gaseous pollutants, the concentration of nitrogen oxides is significantly influenced by the variations in wind speed (23 to 60%) and partially by the variations in temperature (about 17%), relative humidity (2 to 7%), and barometric pressure (2 to 5%).

### Frequency of the occurrence of high pollution loads in ambient air in the study areas

**Fig. 6:**
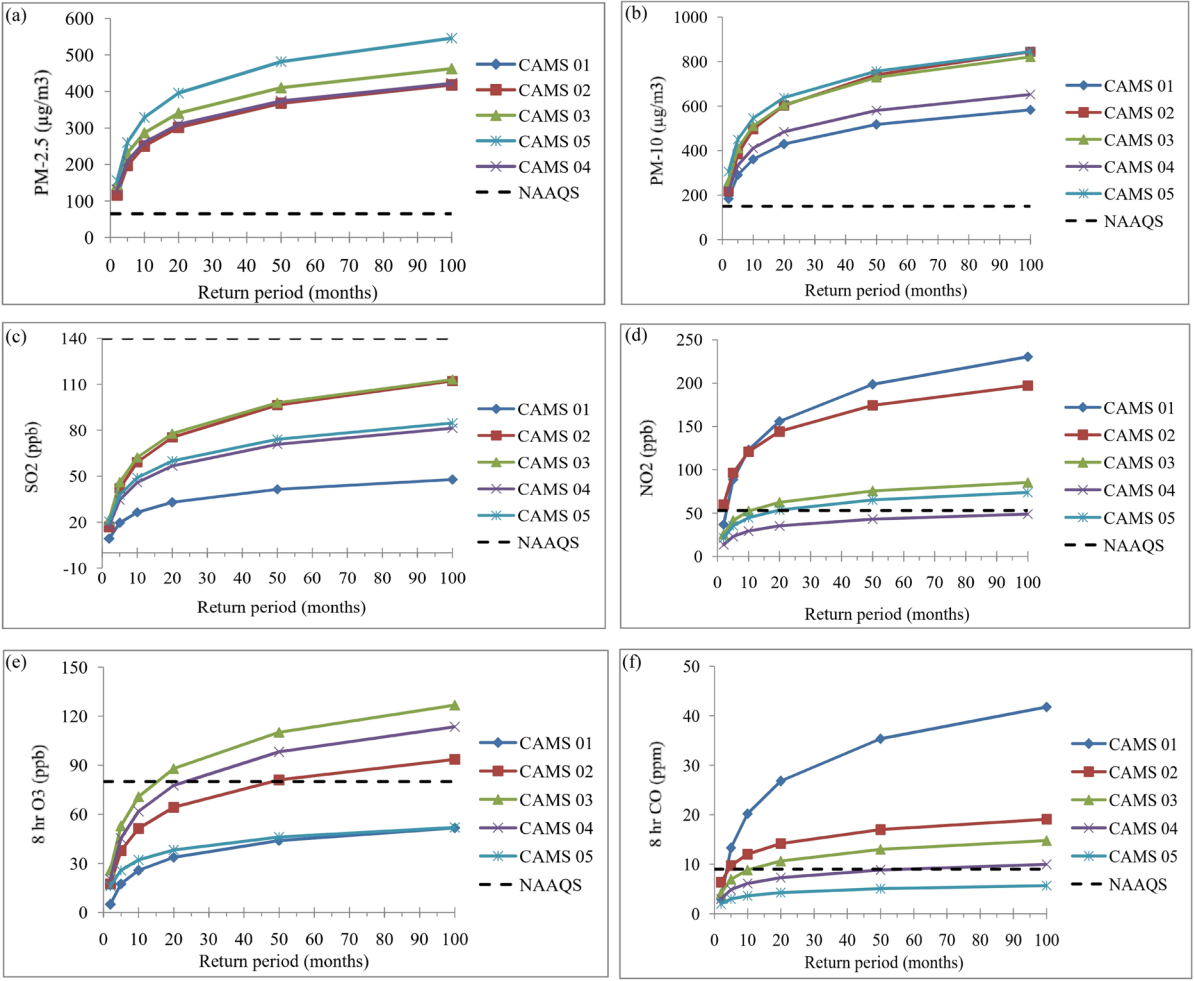
Frequency of the occurrence (in months) of high pollution concentrations at different CAMS stations in Dhaka, Narayangonj, and Gazipur

CAMS 5 at Narayangonj generally showed a relatively higher frequency of high PM_2.5_ and PM_10_ concentrations than the other stations in Dhaka and Gazipur within every specific temporal interval. Among the stations at Dhaka, CAMS 3 at Darus-Salam showed increased chances of exposure to high PM_2.5_ than the other stations. On the other hand, the probability of high pollution loadings for most gaseous pollutants (e.g., NO, NO_2_, SO_2_, CO, and O_3_) is much higher in Dhaka than in Narayangonj and Gazipur (Fig. 6). Therefore, it is apparent that the occupants in those three cities are vulnerable to particulate matter. On the other hand, the likelihood of inhalation of gaseous pollutants might be more prevalent in Dhaka compared to the other cities considered in this study.

### Estimated non-carcinogenic health risk for different age, gender, and occupation groups based on chronic exposures to high pollution levels

The potential non-carcinogenic consequences of exposure to the criteria pollutants were quantified for three age groups (infant, school-going children, and adults), gender group (male and female adults), and occupation group (construction site or field workers). The estimated HQ presents the likelihood of an adverse health outcome, for example, an HQ value higher than 1, indicating possible health hazards (Krupnova et al., 2021).

The data from the Dhaka CAMS locations showed that school-aged children are vulnerable to most pollution parameters. Although all the different classes considered were vulnerable to high particulate matter exposure, the construction or field worker group and the school-aged children were at higher risk than the other groups (Table 3).

**Table 3:** Estimated maximum chronic hazard quotients (HQ) from exposure to polluted air in the study areas

Criteria pollutants	Infants to toddlers up to 3 years age group	School-aged children (6 to 13 years old)	Male adults (>20 years)	Female adults (>20 years)	Construction site/field workers
SO_2_	<1	1.9	<1	<1	2.3
NO_2_	1	175	<1	<1	4.8
O_3_	1	390	1	1	1.5
CO	1	81	1	1	5.6
PM_2.5_	1.8	795	1.9	1.6	324
PM_10_	1.5	524	1.5	1.2	128

**Fig. 7:**
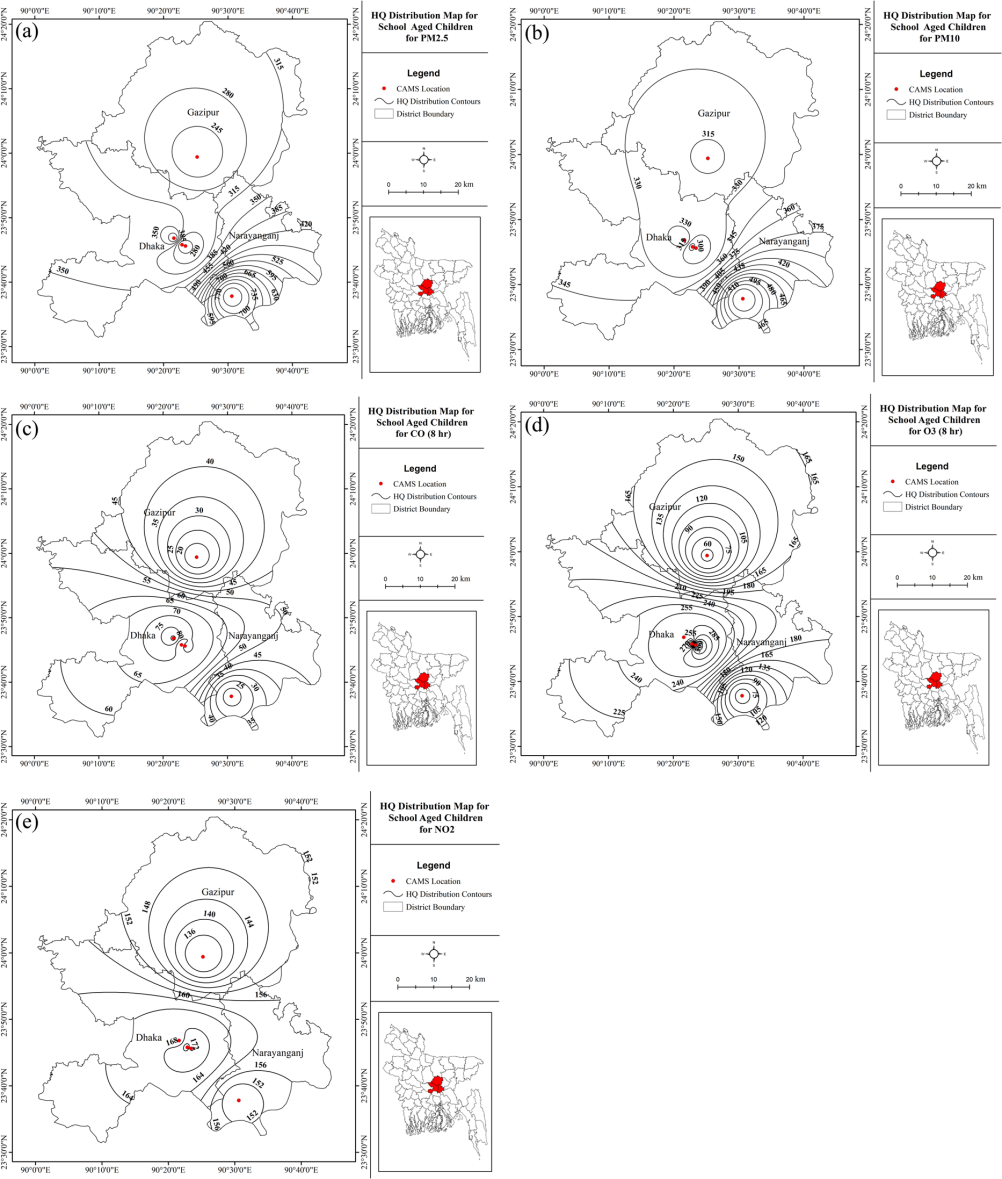
HQ distributions for **a** PM_2.5_, **b** PM_10_, **c** CO, **d** ground-level O_3_, and **e** NO_2_ for the school-aged children in the study areas

**Fig. 8:**
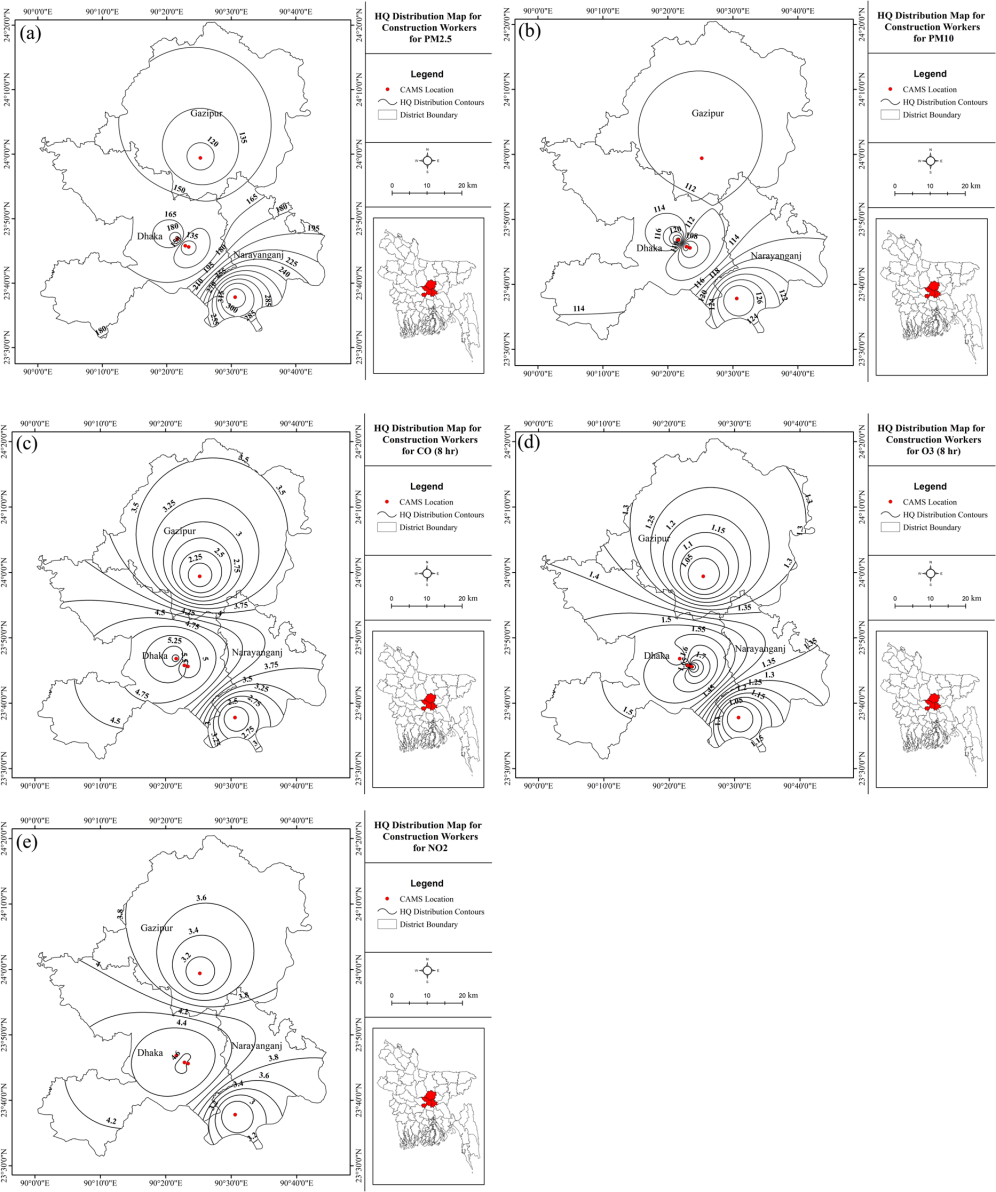
HQ distributions for **a** PM_2.5_, **b** PM_10_, **c** CO, **d** ground-level O_3_, and **e** NO_2_ for the construction site/field workers in the study areas

Among the three areas considered, the highest vulnerability of PM_2.5_ was found in Narayangonj for school-aged children and construction workers (Fig. 7a and Fig. 8a). Like PM_2.5_, the most increased susceptibility of PM_10_ was found in Narayangonj for school-aged children (Fig. 7b and Fig. 8b). Young children are highly vulnerable to ambient air pollution because of their immature immune system, higher air per body weight intake, and underdeveloped lung and metabolic systems (Gouveia & Junger, 2018; Lee, 2021). Additionally, school-going children spend more time playing outdoors, and daily commuting to the schools makes them more exposed than the younger and adult groups to particulates, NO_2_, O_3_, and other gaseous pollutants in the study areas (Fig. 7a–e). The lack of adequate green space, high density of occupants, and placement of the schools near the major roadsides by the private school owners for profit maximization increase the school children’s health risk vulnerability in the study areas. Therefore, these results are consistent with An et al. (2021) and Gabriel et al. (2021) findings. Yang et al. (2021) also found that children aged 4–14 years in 4 cities (Guangzhou, Shanghai, Wuhan, and Xining) in China are highly vulnerable to the adverse health outcomes of PM_2.5_ and NO_2_ exposures. A study carried out by Wu et al. (2022a, b) in Taiwan also reported similar results. Exposure to air particulates and NO_x_ may increase the risks of respiratory diseases, chronic respiratory illness, reduced lung function development, and increased asthma incidence among children in the study areas. Besides, exposures to relatively higher ground-level O_3_ and CO were noticed in Dhaka city, which may also aggravate the vulnerability of increased asthma incidence. These results conform with a study by Dimakopoulou et al. (2020) regarding O_3_ exposure among 10 to 11-year-old children in Greece.

Construction site and field workers of both Dhaka and Narayangonj show high vulnerability to particulate concentrations, almost similar in magnitude (Fig. 8a and b). However, the risks are relatively smaller considering exposure to the gaseous pollutants (Fig. 8c, d, and e). Construction workers, rickshaw pullers, auto rickshaw pullers/compressed natural gas (CNG) drivers, street vendors, and other low-income occupation groups working outdoors are continuously exposed to high particulates, making them vulnerable to cardiovascular and chronic respiratory health risks. These results conform with the results proposed by Maharjan et al. (2022) based on their findings from three major cities in Pakistan, Nepal, and Myanmar. Gandhimathi et al. (2023) in their study also reported similar results.

The male and female adult groups are relatively at less risk, especially those working indoors. Kurata et al. (2020) mentioned that solid fuels for cooking are associated with respiratory illness in girls, especially pregnant women in the rural areas of Bangladesh. Solid fuels for cooking are rare in those urban-centered study areas, except in some informal settlements called slums. However, low-income households sometimes use the same room for cooking and sleeping and use low-quality cooking fuel, which might make the female and the infant groups vulnerable to indoor air pollution and, therefore, increase their health risks to some extent.

### Existing air pollution policies, policy gaps, and scopes for improvement

Bangladesh’s first environmental regulation was proposed as the Environmental Pollution Control Ordinance (EPC) in 1977. Later, several policies and rules were published to control environmental pollution, such as the Environmental Conservation Act (ECA) 1995, Environmental Conservation Rules (ECR) 1997, Brick Kiln Establishment and Installation Act 2013 (2019 amendment), Import Policy Order 2015–2018 2016, National Environmental Policy 2018, Air Pollution Prevention Guidelines 2019, and Air pollution control Rule 2022.

According to ECA (1995), motor vehicles exceeding the standards shall be deemed vehicles emitting harmful fumes or hazardous to health. Later, the government imposed restrictions regarding vehicles emitting smoke injurious to health or the environment not being operated (ECR, 1997). Further, to reduce road dust and emissions from unfit vehicles, the government set several rules; for example, before entering the city from the road or highway, the drivers must clean the wheels of the vehicle, the vendors should not import any car older than four years, and any vehicle declared unfit by the Bangladesh Road and Transport Authority and Dhaka Metropolitan Police should be immediately removed from the road. Additionally, vehicle emission limits were set for Euro-1 for diesel-driven vehicles and Euro-2 for petrol-driven vehicles. Afterward, the government took several initiatives, such as ensuring the circulation and fuel of motor vehicles powered by Euro-3 and Euro-4 model engines. Besides, the Government of Bangladesh decided to ban two-stroke baby taxies, replace them with CNGs, and provide incentives for importing and using eco-friendly (hybrid/electric) vehicles.

To reduce emissions from brick kiln, several policies came into action over time, such as no person shall use any firewood as fuel for burning bricks in a brick kiln. No person shall import and use any coal as fuel containing high sulfur (> 3%), ash, mercury, or similar elements. Considering the level of air pollution in the case of setting up new industrial establishments and brick kilns, degraded air sheds should be avoided. During the transportation of brick or brick kiln raw material, the truck should be covered entirely and transported.

To reduce air pollution from construction activities, many rules and regulations are currently in action. Several include covering building materials (e.g., soil, sand, iron, and cement) inside and outside with a fence and misting the fencing with water at least twice daily to maintain it dust-free. A suitable temporary tent or mattress should be used at the construction site to keep the wind from picking up dust and sand. Avoid throwing construction materials on walkways, roads, or other surfaces; Rajdhani Unnayan Kartripakkha (RAJUK) may impose conditions on air pollution prevention while approving the design of the building. Transportation of construction materials should be covered entirely, and Dhaka Metropolitan Police (DMP) will ensure it. When pitch roads are constructed, open burning of pitch and open burning of agricultural residues should be avoided. A timely plan for road digging for development activities needs to be formulated and implemented by the City Corporation within the stipulated time.

Although there are a lot of policies to reduce air pollution, there are still several important issues that need much more attention, such as the knowledge gap exists in categorizing different industries that cause air pollution. Construction sites cause high pollution, and policies are not implemented very wisely everywhere; the number of unfit motorbikes is increasing dramatically, but most of the bikers do not abide by the emission standards; there is a lack of policies to control private cars; mix mode of the vehicle on the same road increase the traffic jams and therefore burning of fuels for a longer time increase air pollution; there is a lack of proper instruments and skilled personnel for monitoring properly; there is no justification for the weak implementation of air pollution policies and many others.

## Summary of the study

Among the studied locations, a relatively higher frequency of PM_2.5_ and PM_10_ concentrations prevailed at Narayangonj. Moreover, Dhaka city’s population is also exposed to high concentrations of gaseous pollutants, such as nitrogen oxides, CO, and O_3_. Various meteorological factors, such as rainfall, wind speed, wind direction, air temperature, and solar radiation, play critical roles in the variations of pollution concentrations from local and regional sources. A statistical variation of the concentrations of those gaseous pollutants within a 5–10 km distance indicates that the influence of the land use characteristics causes such variability in concentration levels from local sources of pollution. Overall, the increase in the diurnal variability of the concentrations of PMs within a decadal-scale timeframe, increases in the annual duration of high particulate concentrations, and the rise of O_3_ in the cities may simultaneously influence the number and incidences of adverse public health outcomes. School-aged children are vulnerable to the majority of the pollution parameters studied. However, the different population classes considered based on age, gender, and occupation are vulnerable to high particulate exposure, where the construction or field worker group and the school-aged children may be the worst sufferers.

## Conclusions

The concentrations of PM_2.5_ and PM_10_ often exceed the NAAQS in Dhaka, Narayangonj, and Gazipur. The average monthly maximum concentrations of PM_2.5_, PM_10_, NO_2_, O_3_, and CO during the peak dry seasons ranged up to 477 μg/m^3^, 712 μg/m^3^, 210 ppb, 132 ppb, and 79 ppm in the study areas. Out of the many sources of air pollution, fossil fuel burning from vehicles, construction activities, and brick kiln operations are the most significant sources that release high amounts of particulates and gaseous pollutants, especially during the dry season. The possible solutions to reduce pollution from the transportation sources in the study areas might be controlling the vehicle limit, standardizing the vehicles and fuel, practicing penalties for the violation of traffic rules, developing separate lanes for motor vehicles and slow-moving vehicles, encouraging public transport and bicycling, and developing walkable footpaths. In brick manufacturing, we should stop using traditional brick and promote the use of building blocks. Additionally, green technology should be accommodated in the industries that are currently lacking in many industries in and around the cities. For small- to large-scale constructions, we need vigorous enforcement to follow the government’s existing construction codes and a well-equipped strong monitoring government body to monitor the different sources of pollution.

Raising awareness and self-motivation among the public would be one of the most potential interventions to reduce pollution. The number of continuous air monitoring stations needs to be rationally increased to more accurately assess the spatio-temporal variability of the concentrations depending on the major land use characteristics, a significant barrier to making reliable, evidence-based predictions. Therefore, conducting comprehensive assessments by strengthening the air quality monitoring network at a high spatial resolution is crucial, including machine learning-based predictive tools for decision-making and pollution source reduction through improved policy formation and implementation plans. Last, the public health risk assessments using adequate social, health, and environmental datasets should be integrated during the new policy formulation.

## Data Availability

Data sharing is not applicable to this article. The datasets generated during and/or analyzed during the current study are not publicly available but are available from the corresponding author at reasonable request.
